# The association between cold hypersensitivity in the hands and feet and chronic disease: results of a multicentre study

**DOI:** 10.1186/s12906-018-2082-3

**Published:** 2018-01-31

**Authors:** Kwang-Ho Bae, Ho-Yeon Go, Ki-Hyun Park, Ilkoo Ahn, Youngheum Yoon, Siwoo Lee

**Affiliations:** 1 Mibyeong Research Center, Korea Institute of Oriental Medicine, 1672 Yuseong-daero, Yuseong-gu, Daejeon, 34054 Republic of Korea; 2 Department of Korean Internal Medicine, College of Korean Medicine, Se-Myung University, 65 Se-myungro, Jecheon, 27136 Republic of Korea

**Keywords:** Cold hypersensitivity, Flammer syndrome, Cold constitution, Cold disorder, Cold extremities, Primary vascular dysregulation

## Abstract

**Background:**

Cold hypersensitivity in the hands and feet (CHHF) is a common symptom in Korea and patients with CHHF complain of coldness in the hands and feet in an environment that is not considered cold by unaffected people. In traditional East Asian medicine, CHHF is believed to be accompanied by various diseases and symptoms, and is considered a symptom that needs active treatment. CHHF is used for pattern identification in the cold pattern, yang deficiency, and constitution. This study aimed to examine the differences in frequencies of chronic diseases with respect to the presence of CHHF.

**Methods:**

Disease history, CHHF, body measurements, and blood test survey data from 6149 patients collected by 25 medical institutes in Korea were obtained from the Korean Medicine Data Center. The participants were divided into CHHF (*n* = 1909) and non-CHHF groups (*n* = 3017) according to the CHHF survey. The differences in frequencies of 18 diseases were analysed using chi-square tests, and the odds ratios (ORs) for each disease according to CHHF status were examined via logistic regression with adjustment for age, sex, and body mass index (BMI).

**Results:**

Based on chi-square test results, the CHHF group showed a higher frequency of the following diseases: anaemia, hypotension, chronic gastritis, reflux oesophagitis, chronic rhinitis, dysmenorrhoea, and gastroduodenal ulcer. Diseases found in lower frequencies were as follows: hypertension, diabetes mellitus, impaired fasting glucose, dyslipidaemia, stroke, fatty liver, and angina pectoris. In addition, from the logistic regression with adjustment for age, sex, and BMI, the CHHF group showed a lower OR in diabetes mellitus and dyslipidaemia than the non-CHHF group, but a higher OR in degenerative arthritis, chronic gastritis, gastroduodenal ulcer, reflux oesophagitis, and chronic rhinitis.

**Conclusions:**

This study showed that CHHF is associated with chronic disease. Further large-scale prospective studies are needed to validate these associations.

## Background

Cold hypersensitivity in the hands and feet (CHHF) is a sensation of coldness in the hands and feet in an environment not considered cold by unaffected people or a having a heightened cold sensation in a relatively low temperature area. This symptom is relatively common in Korea, especially in women [1, 2].

Although the exact mechanism of CHHF is still unclear, it is associated with a hypersensitive vasoconstrictor response [3, 4] and a heritable phenotype [2]. CHHF symptom presentation is considered important for treatment in traditional East Asian medicine (TEAM) [5]. However, in western medicine, it is perceived as an unremarkable symptom that requires lifestyle management unless it is induced by a specific disease (such as connective tissue disease, peripheral neuropathy, and hypothyroidism) [6].

In TEAM, CHHF is important for identifying yang deficiency and the cold pattern. Furthermore, the book, Internal Classic, by Huangdi introduced the concept of the “spleen and four extremities”, and suggested that the function of the spleen was to control the four limbs and that dysfunction in the limbs may indicate splenic problems [7, 8]. Korean medical doctors believe that CHHF may cause various diseases and symptoms, and can lower the quality of life; therefore, they feel that it requires active treatment [1, 6, 9, 10]. Meanwhile, CHHF has also been associated with an individual’s own constitutional factors (e.g., the “Sasang” constitution, cold and heat pattern) [11, 12].

Studies have examined the association between CHHF and diseases such as functional dyspepsia [5], orthostatic hypotension [13], and dysmenorrhoea [14]. In Japan, studies were conducted focusing on “hie” (cold sensation) or “hiesho” (cold disorder) concepts, which are similar to CHHF, and a few studies have shown an association between cold hypersensitivity and symptoms such as shoulder or neck stiffness and fatigue [10, 15].

However, the concepts behind “hie” and CHHF are not entirely the same; previous studies conducted in Korea had limitations regarding disease, sex, and age of the participants, while a few studies included only a small sample size, thus lacking statistical power [9, 13, 14]. Hence, we believe that further studies are needed to examine their findings. Consequently, we examined the association between CHHF and major chronic diseases using the survey results, body measurement information, and blood test information collected from 25 medical institutes in Korea.

## Methods

### Data collection

**Fig. 1:**
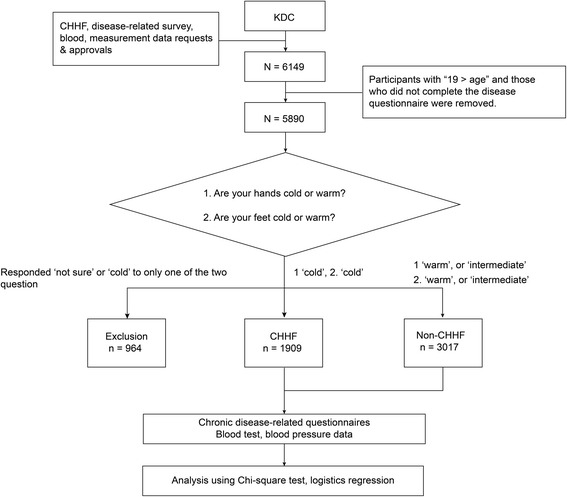
Flow chart of the study. KDC: Korean Medicine Data Centre; CHHF: cold hypersensitivity in the hands and feet; non-CHHF: non-cold hypersensitivity in the hands and feet

This cross-sectional study was conducted based on Korean medical data of 6149 patients collected from 25 Korean medical institutes in Korea between November 2006 and August 2013 and stored at the Korean Medicine Data Center (KDC) [16]. Among these patients, 5890 were aged over 19 years and had complete demographic, blood test results, and body measurements (height, weight, and blood pressure), as well as survey data (CHHF and disease history) (Fig. 1). This study was approved by the Institutional Review Board of KIOM (I-0910/02–001). Consent for participation in the KDC was obtained from each individual.

### Cold hypersensitivity in the hands and feet

Participants completed a questionnaire on their experience of thermal sensations in their hands and feet in the previous six months [5, 17]. In the survey, those who responded “cold” to the questions “Are your hands cold or warm?” and “Are your feet cold or warm?” were classified in the CHHF group (*n* = 1909), and those who responded “warm” or “intermediate” to either question were classified in the non-CHHF group (*n* = 3017). Those who responded, “I don’t know” to either of the two questions (*n* = 964) were excluded from the study (Fig. 1).

### Disease

Chronic diseases that could be confirmed from the disease history survey of KDC data were degenerative arthritis, rheumatoid arthritis, angina, stroke, chronic gastritis, gastroduodenal ulcer, reflux oesophagitis, fatty liver, hepatitis, asthma, chronic rhinitis, and chronic sinusitis. The presence of dysmenorrhoea was confirmed by a menstrual pain scale that is often used in Korean medical clinics [18]. Chronic diseases that could be confirmed by body measurements and blood examination were hypertension, hypotension, impaired fasting glucose (IFG), diabetes mellitus (DM), and dyslipidaemia. The definition and measurement of each disease are as follows.

- Hypertension: Blood pressure was measured from the upper right arm using a sphygmomanometer (FT-500R PLUS, Jawon Medical, Korea). According to the Seventh Joint National Committee on Prevention, Detection, Evaluation, and Treatment of High Blood Pressure (JNC-7), hypertension was defined as a systolic blood pressure (sBP) higher than 140 mmHg or diastolic blood pressure (dBP) higher than 90 mmHg, or a diagnosis of hypertension with blood pressure managed by medication.
- Hypotension: Generally, hypotension is not defined as a disease and has no standard for diagnosis, but there are claims that hypotension accompanies CHHF [10, 13]. Thus, according to the standard mainly used in Korea [19], hypotension is defined as a sBP lower than 90 mmHg or dBP lower than 60 mmHg.
- IFG and DM: Blood analysis was performed using an ADVIA 1800 Auto Analyzer (Siemens, USA). Fasting blood sugar (FBS) was measured after 12 h of fasting. IFG was defined as an FBS between 100 mg/dL and 125 mg/dL, and DM was defined as an FBS higher than 126 mg/dL or a diagnosis of DM with medication for lowering blood sugar, according to the American Diabetes Association.
- Dyslipidaemia: According to the National Cholesterol Education Program Adults Treatment Panel III, dyslipidaemia was defined as a total cholesterol (TC) higher than 240 mg/dL, low-density lipoprotein (LDL) higher than 160 mg/dL, high-density lipoprotein (HDL) lower than 40 mg/dL, triglycerides (TG) higher than 200 mg/dL, or a diagnosis of dyslipidaemia with medication.
- Anaemia: Haemoglobin (Hb) levels were measured using ADIVA2120i (Siemens, USA). According to a standard established by the World Health Organization [20], anaemia was defined as a haemoglobin level lower than 13 g/dL in men and 12 g/dL in women, or a diagnosis of anaemia managed with medication.
- Dysmenorrhoea: Dysmenorrhoea was defined as lower abdominal, back, or pelvic pain occurring just before or during menstruation [21]. However, this study did not distinguish primary and secondary dysmenorrhoea. Among answers of “almost none” (1), “little” (2), “serious” (3), and “very serious” (4) in the menstrual pain questionnaire, answers of “serious” (3) or “very serious” (4) were regarded as indicative of dysmenorrhoea [18, 22].

### Statistical methods

Demographic characteristics of the participants are presented as frequencies and percentages or mean and standard deviation, and analysed using the chi-square test. A comparison of the disease frequency according to CHHF status was presented as frequency and percentage and analysed using the chi-square test. In addition, in order to obtain the odds ratios (OR) and 95% confidence intervals (CIs) for CHHF in each disease, logistic regression with adjustment for sex, BMI, and age was performed. Participants with missing data regarding disease history, blood test results, and body measurement were excluded from the analysis for that respective disease. Furthermore, diabetic participants were excluded from the analysis of IFG, and dysmenorrhoea was analysed only in women aged 19–45 years who still experienced menstruation. The statistical significance level was set at *P* < 0.05. All statistical analyses were conducted using SPSS 21.0 for Windows (IBM Corp., Armonk, NY, USA).

## Results

### Demographic characteristics

The total number of participants was 4926, including 1698 men (34.5%) and 3228 women (65.5%). There were 3017 participants in the non-CHHF group (men, 44.1% women, 55.9%) and 1909 participants in the CHHF group (men, 19.2%; women, 80.8%). Age, height, weight, BMI, sBP, dBP, FBS, TG, TC, LDL, and Hb were significantly higher in the non-CHHF group than the CHHF group, and HDL was significantly higher in the CHHF group than the non-CHHF group (Table 1).

**Table 1:** Participant demographic characteristics and blood test parameters

		Non-CHHF	CHHF	Total	P-value
Sex	Male	1332 (44.1)	366 (19.2)	1698 (34.5)	<0.001
	Female	1685 (55.9)	1543 (80.8)	3228 (65.5)	
Age (y)		48.1 ± 14.0	44.6 ± 13.8	46.8 ± 14.0	<0.001
Height (cm)		163 ± 8.7	161.1 ± 7.5	162.3 ± 8.3	<0.001
Weight (kg)		64.6 ± 11.5	57.6 ± 8.9	61.9 ± 11.1	<0.001
BMI (kg/m^2^)		24.2 ± 3.3	22.2 ± 2.8	23.4 ± 3.3	<0.001
Body temperature (°C)		36.3 ± 0.4	36.3 ± 0.4	36.3 ± 0.4	0.072
Systolic blood pressure		122.8 ± 15.4	117.8 ± 15.6	120.9 ± 15.7	<0.001
Diastolic blood pressure		80.2 ± 11.2	76.7 ± 11.0	78.8 ± 11.2	<0.001
Fasting blood sugar		98.3 ± 27.7	92.6 ± 21.6	96.1 ± 25.6	<0.001
Triglyceride		136.8 ± 96.1	105.1 ± 63.2	124.4 ± 86.1	<0.001
Total cholesterol		187.7 ± 34.5	183.7 ± 33.3	186.2 ± 34.1	<0.001
High-density lipoprotein		48.5 ± 13.0	54.4 ± 13.8	50.8 ± 13.6	<0.001
Low-density lipoprotein		110.7 ± 30.9	105.9 ± 29.8	108.8 ± 30.6	<0.001
Haemoglobin		13.9 ± 1.6	13.2 ± 1.5	13.6 ± 1.6	<0.001

Chi-square test analysis

Results are presented as n (%) or mean ± standard deviation.

*CHHF* cold hypersensitivity in the hands and feet, *BMI* body mass index

### Chi-Square tests analysing the relationship between CHHF and disease

Compared to that in the non-CHHF group, the CHHF group showed a significantly lower frequency of hypertension, DM, IFG, dyslipidaemia, stroke, and fatty liver (*P* < 0.001), as well as angina pectoris (*P* = 0.007). Compared to that in the non-CHHF group, the CHHF group showed a higher frequency of anaemia, hypotension, chronic gastritis, reflux oesophagitis, and chronic rhinitis (*P* < 0.001), as well as dysmenorrhoea (*P* < 0.01) and gastroduodenal ulcer (*P* < 0.05). There was no significant difference in degenerative arthritis, rheumatoid arthritis, asthma, and chronic sinusitis between the two groups (Table 2).

**Table 2:** Disease frequencies in the CHHF and non-CHHF groups

		Non-CHHF	CHHF	Total	*P*-value
Anaemia	No	2650 (89.6)	1608 (84.5)	4258 (87.6)	< 0.001
	Yes	306 (10.4)	296 (15.5)	602 (12.4)	
Angina pectoris	No	2926 (97.0)	1875 (98.2)	4801 (97.5)	0.007
	Yes	91 (3.0)	34 (1.8)	125 (2.5)	
Asthma	No	2891 (95.8)	1829 (95.8)	4720 (95.8)	0.980
	Yes	126 (4.2)	80 (4.2)	206 (4.2)	
Chronic gastritis	No	2683 (88.9)	1537 (80.5)	4220 (85.7)	< 0.001
	Yes	334 (11.1)	372 (19.5)	706 (14.3)	
Chronic rhinitis	No	2739 (90.8)	1664 (87.2)	4403 (89.4)	< 0.001
	Yes	278 (9.2)	245 (12.8)	523 (10.6)	
Chronic sinusitis	No	2879 (95.4)	1799 (94.2)	4678 (95.0)	0.063
	Yes	138 (4.6)	110 (5.8)	248 (5.0)	
Degenerative arthritis	No	2731 (90.5)	1714 (89.8)	4445 (90.2)	0.397
	Yes	286 (9.5)	195 (10.2)	481 (9.8)	
Diabetes mellitus	No	2683 (90.8)	1815 (95.4)	4498 (92.6)	< 0.001
	Yes	272 (9.2)	87 (4.6)	359 (7.4)	
Dyslipidaemia	No	1722 (58.3)	1437 (75.6)	3159 (65.0)	< 0.001
	Yes	1234 (41.7)	465 (24.4)	1699 (35.0)	
Dysmenorrhoea	No	422 (78.9)	561 (72.6)	983 (75.2)	0.009
	Yes	113 (21.1)	212 (27.4)	325 (24.8)	
Fatty liver	No	2737 (90.7)	1817 (95.2)	4554 (92.4)	< 0.001
	Yes	280 (9.3)	92 (4.8)	372 (7.6)	
Gastroduodenal ulcer	No	2896 (96.0)	1806 (94.6)	4702 (95.5)	0.023
	Yes	121 (4.0)	103 (5.4)	224 (4.5)	
Hypertension	No	1992 (66.5)	1460 (76.5)	3452 (70.4)	< 0.001
	Yes	1004 (33.5)	449 (23.5)	1453 (29.6)	
Hypotension	No	2824 (94.3)	1733 (90.8)	4557 (92.9)	< 0.001
	Yes	172 (5.7)	176 (9.2)	348 (7.1)	
Impaired fasting glucose	No	2050 (91.6)	1749 (96.4)	4206 (93.5)	< 0.001
	Yes	633 (8.4)	66 (3.6)	292 (6.5)	
Reflux oesophagitis	No	2733 (90.6)	1626 (85.2)	4359 (88.5)	< 0.001
	Yes	284 (9.4)	283 (14.8)	567 (11.5)	
Rheumatoid arthritis	No	2958 (98.0)	1860 (97.4)	4818 (97.8)	0.154
	Yes	59 (2.0)	49 (2.6)	108 (2.2)	
Stroke	No	2700 (89.5)	1795 (94.0)	4495 (91.3)	< 0.001
	Yes	317 (10.5)	114 (6.0)	431 (8.7)	

Chi-square test analysis

*P*-values were calculated from chi-square tests comparing the CHHF with the non-CHHF groups

*CHHF* cold hypersensitivity in the hands and feet

### Odds ratios for disease according to CHHF status

**Fig. 2:**
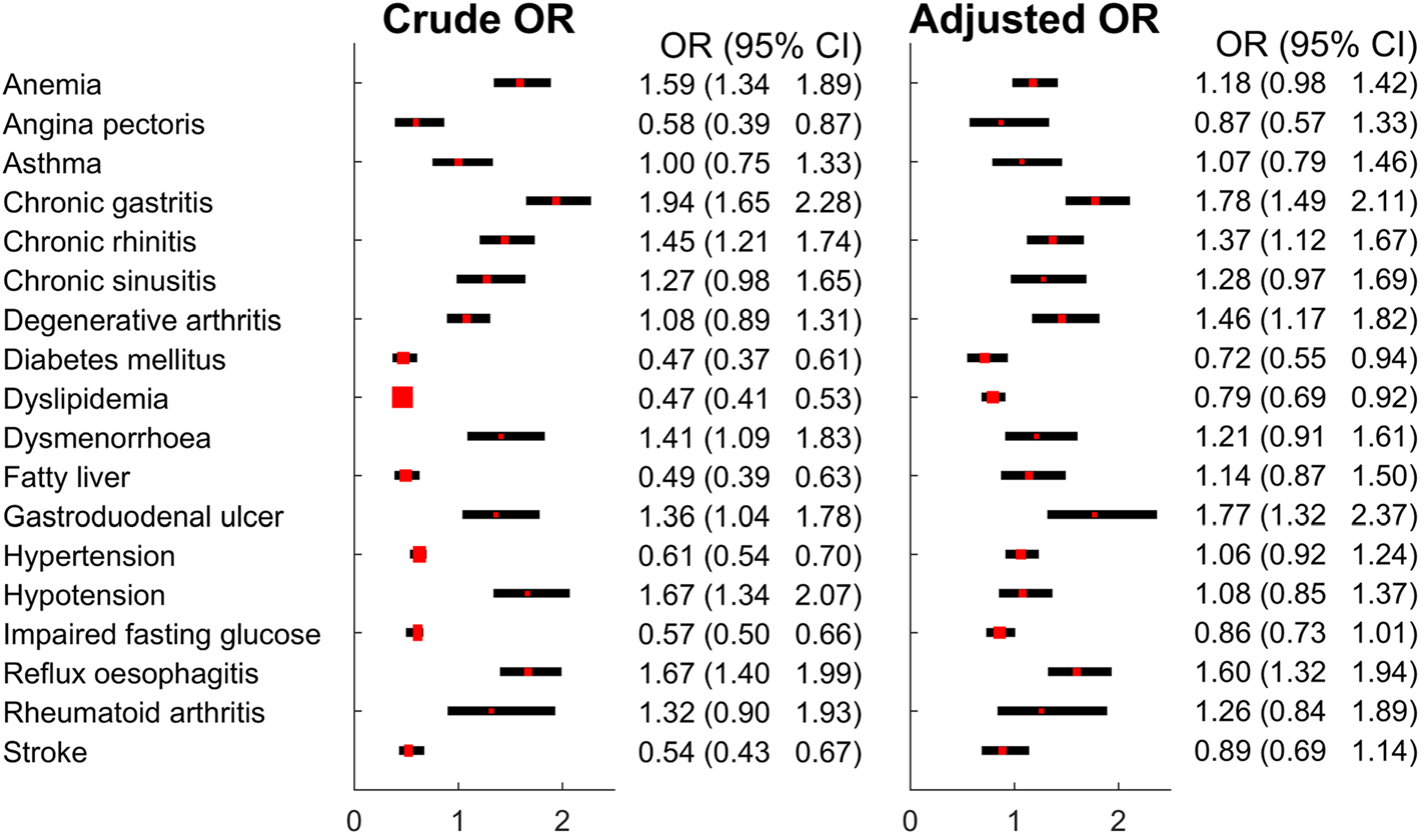
The odds ratios (ORs) and 95% confidence intervals (CIs) for diseases according to CHHF status. Logistic regression analysis, adjusted for sex, age, and body mass index. CHHF: cold hypersensitivity in the hands and feet; ref.: reference. “Non-CHHF” was employed as the reference in every analysis

Figure 2 shows the ORs and 95% confidence intervals (CI) for each disease in the CHHF group with non-CHHF as a reference. Without adjustment, ORs lower than 1 were observed in hypertension, DM, dyslipidaemia, IFG, angina, stroke, and fatty liver, and ORs higher than 1 were observed in hypotension, anaemia, chronic gastritis, gastroduodenal ulcer, reflux oesophagitis, and chronic rhinitis.

With adjustments for age, sex, and BMI, ORs lower than 1 were observed in DM and dyslipidaemia, and ORs higher than 1 were observed in degenerative arthritis, chronic gastritis, gastroduodenal ulcer, reflux oesophagitis, and chronic rhinitis (Fig. 2).

## Discussion

CHHF is not only inconvenient for individuals in their daily life, but is important for pattern identification (such as the cold pattern and yang deficiency) in TEAM. In TEAM, pattern identification is an important factor to consider during disease diagnosis when prescribing herbal medicine, so it is important that both be identified [23]. Moreover, while many people experience CHHF and it has been considered a serious symptom in East Asia for a long time, it is not well-known if CHHF is associated with various diseases [2, 24]. Thus, clarifying CHHF and potential associated diseases is crucial [8, 15]; however, this has not been studied using a large sample size. Therefore, this study aimed to determine whether CHHF is associated with various diseases.

Previous studies on CHHF were focused on a single disease or on the association between other symptoms and CHHF. Furthermore, studies on “hie” (cold disorder), which is a concept similar to CHHF in Japan, examined the association between cold disorder and various symptoms, but did not focus on its association with other diseases [10, 15]. Unlike previous studies that examined the association of CHHF with a single disease or other symptoms, this study analysed its association with various diseases. Our study results supported some of the findings from previous studies and the TEAM literature, but also produced some unique findings.

When participant age, sex, and BMI were adjusted, the CHHF group showed ORs higher than 1 in degenerative arthritis, chronic gastritis, gastroduodenal ulcer, reflux oesophagitis, and chronic rhinitis compared to those in the non-CHHF group. This result is in line with a previous report showing a higher frequency of functional dyspepsia in the CHHF group [5] and a report showing higher frequency of atrophic gastritis in yang deficiency and the cold pattern with the index of CHHF, suggesting that the frequency of organic gastric diseases (chronic gastritis, gastroduodenal ulcer, and reflux oesophagitis) is high in CHHF. There are several reports showing that degenerative arthritis and chronic rhinitis are more common in the cold pattern than the heat pattern [25, 26]. However, reflux oesophagitis is more common in the heat pattern according to TEAM, which was different from our study result [27]. In fact, there are currently no theories or research findings that can clearly explain the correlation or the causal relationship between CHHF and these diseases. However, based on the results of a few studies, it can be conjectured that local or systemic hypoxic effects may arise as interference with blood circulation in certain vulnerable organs occurs, similar to the observation of poor peripheral blood circulation in the hands and feet; these effects may be involved in the pathogeneses of various diseases [24, 28]. Endothelin-1 and autonomic dysfunction, which are known to be involved in poor peripheral blood circulation, may partially affect the pathogenesis, persistence, and worsening of a disease [29–36].

In our study, DM and dyslipidaemia had ORs lower than 1 in the CHHF group compared to those in the non-CHHF group when adjustments were made for age, sex, and BMI. This is contradictory to the results of a previous study showing that diabetic peripheral neuropathy, Type-I DM, and atherosclerosis could be the causes of CHHF [6, 10, 37]. However, our results seem plausible as CHHF is not just a simple symptom but can be used to differentiate cold/heat patterns and the “Sasang” constitution. It is reported that the cold or yang deficiency pattern has the characteristics of hypofunction and a reduction in metabolic activity while the heat pattern has characteristics of hyperfunction, an increased urge to drink, a flushed face, and irritability [7, 38]. In addition, the Sasang typology, which is a branch of traditional Korean personalized medicine, classifies humans into four types based on differences in functional activity among the four viscera. The So-Eum type, which is one of these four types, involves a loss of splenic function, and a high rate of CHHF [12, 39]. In addition, Park’s study suggested that CHHF could increase levels of circulating adiponectin and lower the risk of metabolic syndrome [17]. This suggests that CHHF may serve as the basis for explaining why the ORs of DM and dyslipidaemia were lower in this study. Moreover, Konieczka’s study suggests that Flammer syndrome (a combination of symptoms and signs that result from a predisposition to an increased sensitivity in general) may have an advantage in protecting from atherosclerosis [40]. Thus, we believe that CHHF can be associated not only with a high frequency of certain diseases, but also with a low frequency of them.

High ORs for dysmenorrhoea and hypotension were observed in the CHHF group, similar to the results of a previous study, showing that CHHF and the cold pattern are common in patients with dysmenorrhoea, and that the frequency of CHHF is high in orthostatic hypotension [13–15, 40].

CHHF is a commonly observed symptom in East Asian countries including Korea and presumed to occur because of a genetic disposition and imbalance of the autonomic nerves triggering impaired peripheral circulation [2, 3, 41]. However, the true cause has still not been determined. It is known that sex and BMI largely influence the frequency of CHHF and are associated with several symptoms and diseases [5, 10, 13–15, 42–44]. Our study examined the association of CHHF with several diseases, allowing a better understanding of CHHF pathogenesis.

This study has a few limitations. First, because it was a cross-sectional study, a causal relationship cannot be examined. Thus, it is unknown if our results were the effects of disease, cause of disease, or caused by the treatment of disease. Second, bias may have existed because most disease data were obtained via surveys and thus dependent on patient memory, and patients underwent only one round of examinations and measurements. Dysmenorrhoea was not classified into primary and secondary types, and the validity and reliability of the questionnaire used in this study have not been verified. In addition, we confirmed whether the participants had CHHF or not based on the sensations they experienced in the last six months. However, considering that thermal sensations are affected by ambient temperature, it is necessary to maintain a constant temperature inside the measurement room of each institution [4]. Unfortunately, we could not confirm if the ambient temperature was constantly maintained in the measurement room in our study.

Prospective studies with accurate disease diagnoses, which also allow the exploration of a causal relationship, should be conducted in the future.

## Conclusions

In this study, we found that CHHF was associated with DM, dyslipidaemia, degenerative arthritis, chronic gastritis, gastroduodenal ulcer, reflux oesophagitis, and chronic rhinitis. Our results allow a better understand of CHHF pathogenesis.

## Abbreviations

BMI: Body mass index
CHHF: Cold hypersensitivity in the hands and feet
CIs: Confidence intervals
dBP: Diastolic blood pressure
DM: Diabetes mellitus
FBS: Fasting blood sugar
Hb: Haemoglobin
HDL: High-density lipoprotein
IFG: Impaired fasting glucose
LDL: Low-density lipoprotein
ORs: Odds ratios
sBP: Systolic blood pressure
TC: Total cholesterol
TEAM: Traditional East Asian medicine
TG: Triglyceride
